# Alarm or emotion? intranasal oxytocin helps determine information conveyed by dog barks for adult male human listeners

**DOI:** 10.1186/s12862-024-02198-2

**Published:** 2024-01-14

**Authors:** Péter Pongrácz, Csenge Anna Lugosi, Luca Szávai, Atina Gengeliczky, Nikolett Jégh-Czinege, Tamás Faragó

**Affiliations:** 1 Department of Ethology, ELTE Eötvös Loránd University, Pázmány Péter sétány 1/c, Budapest, 1117 Hungary; 2 Neuroethology of Communication Lab, Department of Ethology, Eötvös Loránd University, Budapest, Hungary

**Keywords:** Interspecific communication, Emotions, Attention-eliciting, Dog, Human, Oxytocin, Empathy, Alarm

## Abstract

**Background:**

Barks play an important role in interspecific communication between dogs and humans, by allowing a reliable perception of the inner state of dogs for human listeners. However, there is growing concern in society regarding the nuisance that barking dogs cause to the surrounding inhabitants. We assumed that at least in part, this nuisance effect can be explained by particular communicative functions of dog barks. In this study we experimentally tested two separate hypotheses concerning how the content of dog barks could affect human listeners. According to the first hypothesis, barks that convey negative inner states, would especially cause stress in human listeners due to the process called interspecific empathy. Based on the second hypothesis, alarm-type dog barks cause particularly strong stress in the listener, by capitalizing on their specific acoustic makeup (high pitch, low tonality) that resembles to the parameters of a baby’s cry. We tested 40 healthy, young adult males in a double-blind placebo controlled experiment, where participants received either intranasal oxytocin or placebo treatment. After an incubation period, they had to evaluate the (1) perceived emotions (happiness, fear and aggression), that specifically created dog bark sequences conveyed to them; and (2) score the annoyance level these dog barks elicited in them.

**Results:**

We found that oxytocin treatment had a sensitizing effect on the participants’ reactions to negative valence emotions conveyed by dog barks, as they evaluated low fundamental frequency barks with higher aggression scores than the placebo-treated participants did. On the other hand, oxytocin treatment attenuated the annoyance that noisy (atonal) barks elicited from the participants.

**Conclusions:**

Based on these results, we provide first-hand evidence that dog barks provide information to humans (which may also cause stress) in a dual way: through specific attention-grabbing functions and through emotional understanding.

**Supplementary Information:**

The online version contains supplementary material available at 10.1186/s12862-024-02198-2.

## Background

Domestication is an evolutionary process that usually results in a well described set of phenotypical traits [1] that separate domesticated species from their wild relatives. In case of the dog (*Canis familiaris*), the oldest domesticated animal (e.g., [2]), the most typical species-specific features are those socio-cognitive and behavioral phenotypes that enable the dog to coexist with humans in an intricately complex system of dependency [3]. Among these, preference towards humans over conspecifics [4], attachment to the owner [5], human-directed referencing [6], and cooperating with humans rather than with other dogs [7], all manifest in dogs much more readily, than in tame specimens of their closest relatives, the gray wolf (*Canis lupus*). Dogs also show a wide array of communicative skills that seem to be honed towards understanding various human signals (visual, [8]; acoustic, [9]; and olfactory [10–11]. Dogs themselves have particular communicative features that could show the effect of domestication, one of the more notable ones is their most abundant type of vocalization: barking [12].

Dog barks show unique features compared to the barks of wolves. Beyond the obvious quantitative difference (dogs bark more, [12]), most remarkably dog barks became acoustically diverse, covering such contextual diversity that in wolves would be covered by other sorts of vocalizations (such as growling or howling, [12]). According to one of the explanatory theories [13–14], dog barks became the main acoustic signal type that dogs ‘use’ towards a new ‘audience’: humans. It was found that barks convey reliable information about the inner state of the dog [15]; as well as contextual information [16] for human listeners. Compared to the predominantly low-pitched and noisy (atonal) barks of wolves [17], dog barks show a more variable acoustic nature. It was found that for humans, the combination of fundamental frequency, harmonic-to-noise ratio (tonality) and pulse of the barking (the length of inter-bark intervals) all carry emotional information. Deep, noisy and fast pulsing barks are thought to belong to an aggressive dog, while-high pitched, tonal and slow barks convey fear and despair. Other combinations of these three parameters may convey playfulness and happiness [15]. These effects are rather robust, and they work similarly in children and adults [18], independent of dog-related experiences [16], or cultural background [19].

Vocalizations across a wide selection of mammalian (and avian) species have a conservative and highly similar nature in how they encode the inner state of the signaler. The explanation for this is two-fold. From the aspect of signal evolution, according to the structural-functional ‘rule’ of Morton [20], particular acoustic parameters became typical for the emitter of a given signal because the anatomical features of the individual (including its vocal apparatus) were highly predictive for its likely intentions. For example, larger individuals were likely to be aggressive, and the larger body and larger vocal apparatus were likely to produce deep and noisy vocalizations. Small individuals were likely to signal submission and lack of aggression in case of conflict, and their smaller vocal organs were more likely emitting higher pitched, cleaner vocalizations. Besides Morton’s theory, the so-called ‘source-filter’ theory of acoustic production, provides a mechanistic explanation for the similarities of how the vocalizations of various species can have similarly encoded indexical and emotional content [21]. The source–filter theory states that vocal signals result from a two-stage production, with the glottal wave generated in the larynx (the source), being subsequently filtered in the supralaryngeal vocal tract (the filter). Physiological fluctuations in emotional or motivational state have been found to influence the acoustic characteristics of signals in a reliable and predictable manner. As the innervation of the production and filtering components of the vocal tract shows high similarity across mammals [22], this explains how particular affective states will be expressed in a similar acoustic way in various species.

Although the theory of domestication-related changes in the dog’s vocal output [13–14] suggests that the qualitative and quantitative proliferation of dog barks would serve a more effective communication of the dogs’ inner states towards humans with basically the same type of vocalization, there are increasing parallel concerns about the negative effect of dog barks on the coexistence of the two species. Nuisance barks represent a worldwide concern [23], resulting in anti-dog keeping legislation at the community level [24], relinquishment of dogs [25], anti-barking interventions that can range from training [26] to punitive devices [27] and the controversial process of surgical de-barking of the offending dogs [28]. It would be easy to consider dog barks as being just one more component of noise pollution, where barks would be annoying because they are too loud, emitted too abundantly, or in the wrong time [29], but that is only part of the reason we find dog barks annoying.

Related to the previously detailed communicative function of barking towards humans, recently we proposed a new theory that focuses on particular acoustic components that could make particular bark types more annoying than others [30]. While we acknowledge that to a certain extent every dog bark type represents a rather unpleasant acoustic experience to human listeners (which fits with the assumption that dog barks can serve as mobbing signals, [31], according to our ‘*communicative relevance of nuisance barks’* theory [30], humans would become more annoyed by those barks that have a high attention-eliciting effect because of their particular communicative content. We found that barks which convey negative emotions (aggression, fear, despair) would cause stronger nuisance for the humans than barks with ‘positive’ emotional content. In a follow-up study, it was also shown that there is a specific combination of fundamental frequency and tonality that was especially annoying to human listeners [32]. As these high-pitched and noisy dog vocalizations were acoustically similar to babies’ crying, and young (reproductive age) adults reacted the strongest to them, we hypothesized that there was a past selective emergence of an especially effective attention-eliciting type of bark. According to this theory, barks mostly become annoying if the human listener cannot intervene, which eventually leads to frustration [32]. It is important to see that we do not propose that dog barks were evolved to be ‘annoying’ for humans. Dog barks convey vital information about the dynamically changing inner state of the signaler to human listeners and the acoustic variability of dog barks that enables this function can be regarded as a new feature related to domestication [14]. Barking of the wolf only convey agonistic content, and other inner states are expressed with other types of vocalizations [12]. In contrast, dogs can express a wide array of inner states with barking only (from happiness to fear), and the acoustic characteristics of dog barking changed to a much more variable phenotype [33] compared to the generally low-pitched and noisy wolf barks.

This leads to two parallel theories that could explain why dog barks elicit annoyance in humans. One of these theories (i) focuses on affective communication [30], where humans can read the inner state-related information in dog barks [15], and mostly the perceived negative emotions elicit nuisance in the receivers. This mechanism could be explained on the basis of inter-specific empathy [34], which has an important role in dog-human interactions [19]. The other explanation (ii) is that specific barks have a strong attention-eliciting effect [32] and just like baby cries, in case of prolonged exposure they may elicit stress and eventually frustration and anger from the listeners [35]. As the attention-eliciting and affective content of dog barks would be hard to disentangle acoustically, or with behavioral tests alone, in this study we opted for applying intranasal oxytocin treatment to the human participants with the aim of getting a clearer picture of how the mechanisms of particular dog barks affect humans.

The neuropeptide hormone oxytocin has a complex and widespread effect in the body [36], and in this study we will focus only on its mediating effect on emotional understanding (as a ‘central’ effect, influencing affective empathy, [37]) and its attenuating effect on psycho-social stress reactions (as a ‘peripheral’ effect, e.g., [38]. There are many indications that oxytocin has a positive effect on trusting others [39] and recognizing other humans’ emotions (e.g., facial expressions, [40]). In that double-blind study [40], with the use of fMRI technology, it was found that intranasal oxytocin suppressed the right hemisphere’s amygdale activation, thereby reducing the participants’ fear reactions towards angry and frightened human faces. Kosfeld and colleagues [41] found that oxytocin takes a role in the formation of positive, prosocial behavioral patterns, what they considered as of fundamental importance in the formation of ‘trust’ (“an individual’s willingness to accept social risks arising through interpersonal interactions”). Although Singer and colleagues [42–43] did not find direct association between intranasally administered oxytocin and the neural mechanisms responsible for emotional distress, it was later found that the polymorphism of the OXTR gene shows an association with emotional empathy [44]. There are numerous studies either showing supporting evidence of the connection between oxytocin and the participants’ affective empathy performance (e.g., [45]) or the lack of such associations (e.g., [46]).

Regarding stress-attenuation, oxytocin has a negative effect on cortisol levels in the case of physical exercise [47] and social stress [48]. Interestingly, it was found that emotional support and oxytocin together had the strongest stress-attenuation, probably because positive human interactions themselves enhance oxytocin production [49]. It is worth mentioning at the same time that recently some authors did not find connection between the effect of particular stress-reducing mental training methods and their connection with modulating the stress-induced acute plasma oxytocin release, and they emphasize the need of further investigations [50].

### Goals, hypotheses, predictions

In this study we wanted to find out whether dog barks affect human listeners predominantly through their emotional content (affective inner state communication), or because they evoke attention from the listeners (‘alarm calls’). Because we previously found that male participants were more annoyed by barking dogs [30], and young adults responded most intensely to nuisance barks [32], we tested only young men in a double-blind, placebo controlled experiment with intranasal oxytocin administration. As we mentioned previously, researchers so far did not arrive to an unambiguous consensus regarding the exact effect of intranasally administered oxytocin on affective empathy and mediating social stress. In the framework of the present study, we assumed an overall positive effect of oxytocin on the participants’ affective empathy, and we assumed an attenuating effect of oxytocin on the participants’ reactions to the attention-grabbing (‘alarm’) vocalizations. According to our *first hypothesis*, nuisance barks [30, 51] cause stress through their unique acoustic structure [32, 52], thus we predicted that intranasal oxytocin, through its stress-attenuator effect [53], would lessen the elicited nuisance in the listeners. Our *second hypothesis* considered that the oxytocin would affect how participants would react to the emotional content of dog barks. As there are indications that oxytocin has an effect on affective empathy (emotional understanding) [54–55], here we predicted that intranasal oxytocin would modify the participants’ reactions to particular (especially the negative valence) dog barks in the playback study.

## Results

**Fig. 1:**
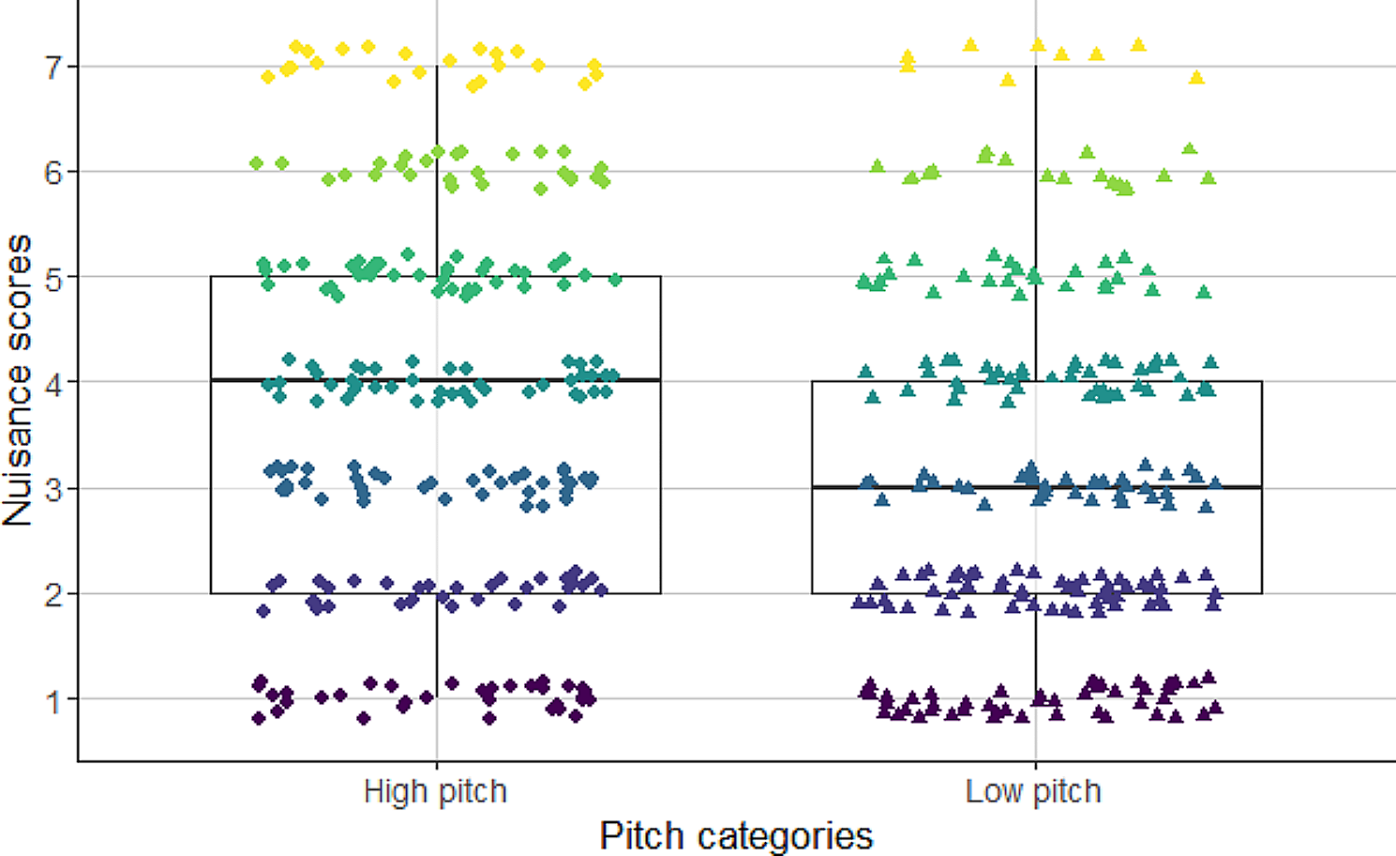
The effect of fundamental frequency on the assessment of the annoyance-level of dog barks. High fundamental frequency barks were found to be more annoying by the subjects independent of the treatment type

**Fig. 2:**
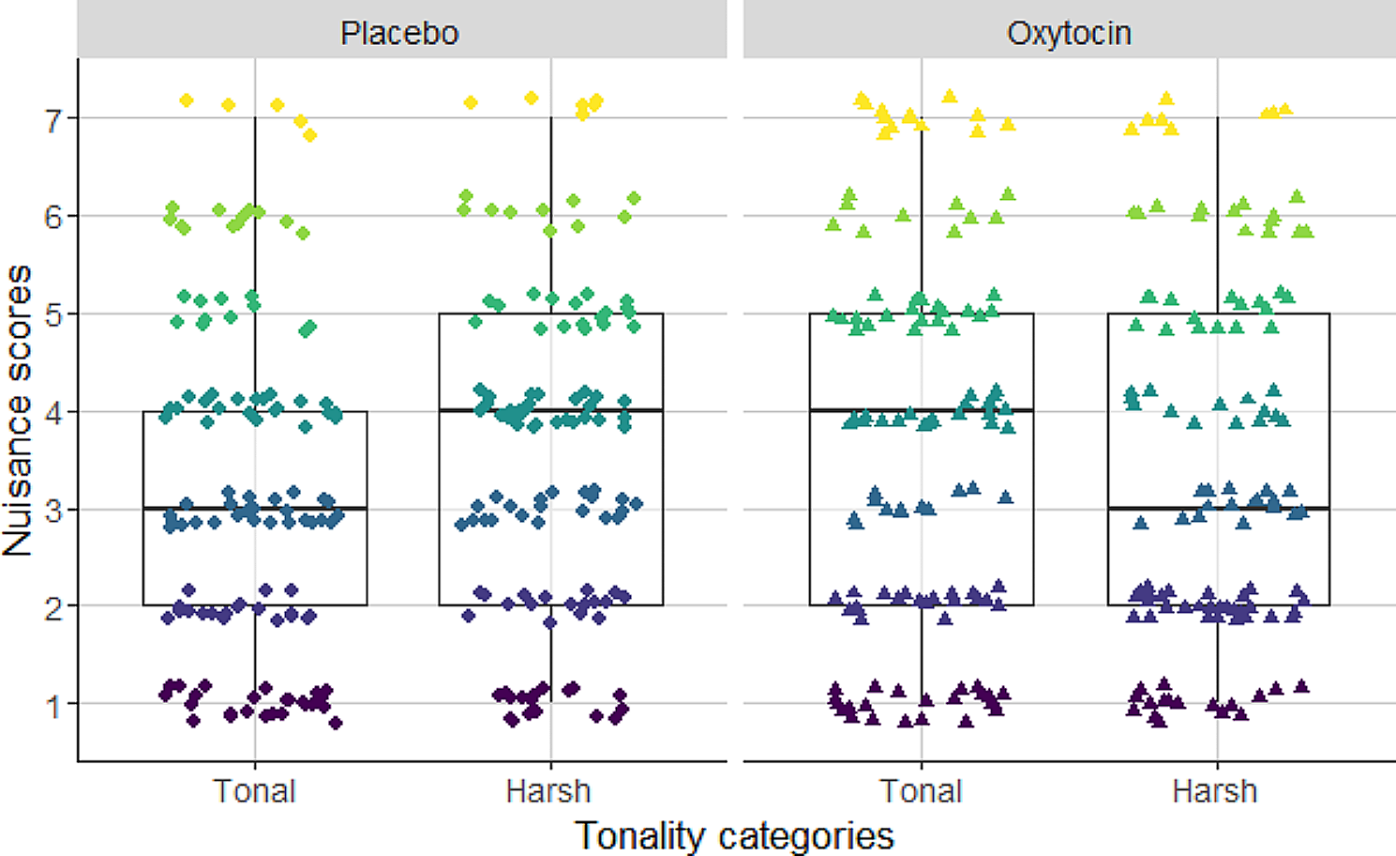
Two-way interaction between tonality and treatment. Participants who received placebo treatment found atonal barks more annoying than tonal barking. However, participants who received oxytocin treatment, showed no significant difference between the two tonality categories

‘Annoyance’ scores showed a significant association with the fundamental frequency of barks. The higher was the pitch of the barks, the participants considered it as being more annoying than dogs barking in a low pitch (see Table 1; Fig. 1). Furthermore, we found significant interaction between the tonality of barks and the treatment of subjects (see Table 2; Fig. 2). With Tukey’s post hoc test we found that participants who received placebo treatment rated the low tonality (noisy) barks more annoying than high tonality (clear) barks (cum. prob ± SE = 0.056 ± 0.001; z = 47.542; *p* < 0.001; Fig. 1). In the case of participants receiving the oxytocin treatment we did not find significant difference between the annoyance scores of noisy and tonal barks (cum. prob ± SE=-0.039 ± 0.033; z=-1.177; *p* = 0.239).

**Table 1:** Fundamental frequency as main effect and two-way interaction between tonality and treatment

	Model 1: Annoyance scores			
*Predictors*	*Odds Ratios*	*CI*	*Statistic*	*p*
pitch [Low]	0.428	0.426–0.430	-346.883	**< 0.001**
tonality [Noisy]	1.516	1.509–1.523	169.717	**< 0.001**
Treatment [Oxytocin]	1.748	0.801–3.818	1.402	0.161
tonality [Noisy] × Treatment [Oxytocin]	0.497	0.311–0.796	-2.909	**0.004**
**Random Effects**				
σ^2^	3.29			
τ_00 ID_	2.51			
ICC	0.43			
N _ID_	40			
Observations	480			
Marginal R^2^ / Conditional R^2^	0.037 / 0.454			

**Table 2:** Amount of oxytocin in IU (International Unit) used during intranasal treatment in scientific literature

Authors of article, date	Administered dose
[66] Ditzen B, Schaer M, Gabriel B, Bodenmann G, Ehlert U, Heinrichs M. Intranasal oxytocin increases positive communication and reduces cortisol levels during couple conflict. Biol Psychiatry. 2009;65:728 − 31.	40 IU
[67] Bartz J, Simeon D, Hamilton H, Kim S, Crystal S, Braun A, et al. Oxytocin can hinder trust and cooperation in borderline personality disorder. Soc Cogn Affect Neurosci. 2010;nsq085.	40 IU
[68] Domes G, Lischke A, Berger C, Grossmann A, Hauenstein K, Heinrichs M, Herpertz SC. Effects of intranasal oxytocin on emotional face processing in women. Psychoneuroendocrinology. 2010;35:83–93.	24 IU
[69] Guastella AJ, Einfeld SL, Gray KM, Rinehart NJ, Tonge BJ, Lambert TJ, Hickie IB. Intranasal oxytocin improves emotion recognition for youth with autism spectrum disorders. Biol Psychiatry. 2010;67:692-4.	18 and 24 IU
[70] De Dreu CK, Greer LL, Van Kleef GA, Shalvi S, Handgraaf MJ. Oxytocin promotes human ethnocentrism. Proc Natl Acad Sci. 2011;108: 1262-6.	24 IU
[71] Rilling JK, DeMarco AC, Hackett PD, Thompson R, Ditzen B, Patel R, Pagnoni G. Effects of intranasal oxytocin and vasopressin on cooperative behavior and associated brain activity in men. Psychoneuroendocrinology. 2012;37:447 − 61.	20 and 24 IU
[72] Kis A. Kemerle K, Hernádi A, Topál J. Oxytocin and social pretreatment have similar effects on processing of negative emotional faces in healthy adult males. Front Psychol. 2013;4:532.	24 IU
[73] Palgi S, Klein E, Shamay-Tsoory SG. Intranasal administration of oxytocin increases compassion toward women. Soc Cogn Affect Neurosci. 2015;10:311-7.	24 IU

**Fig. 3:**
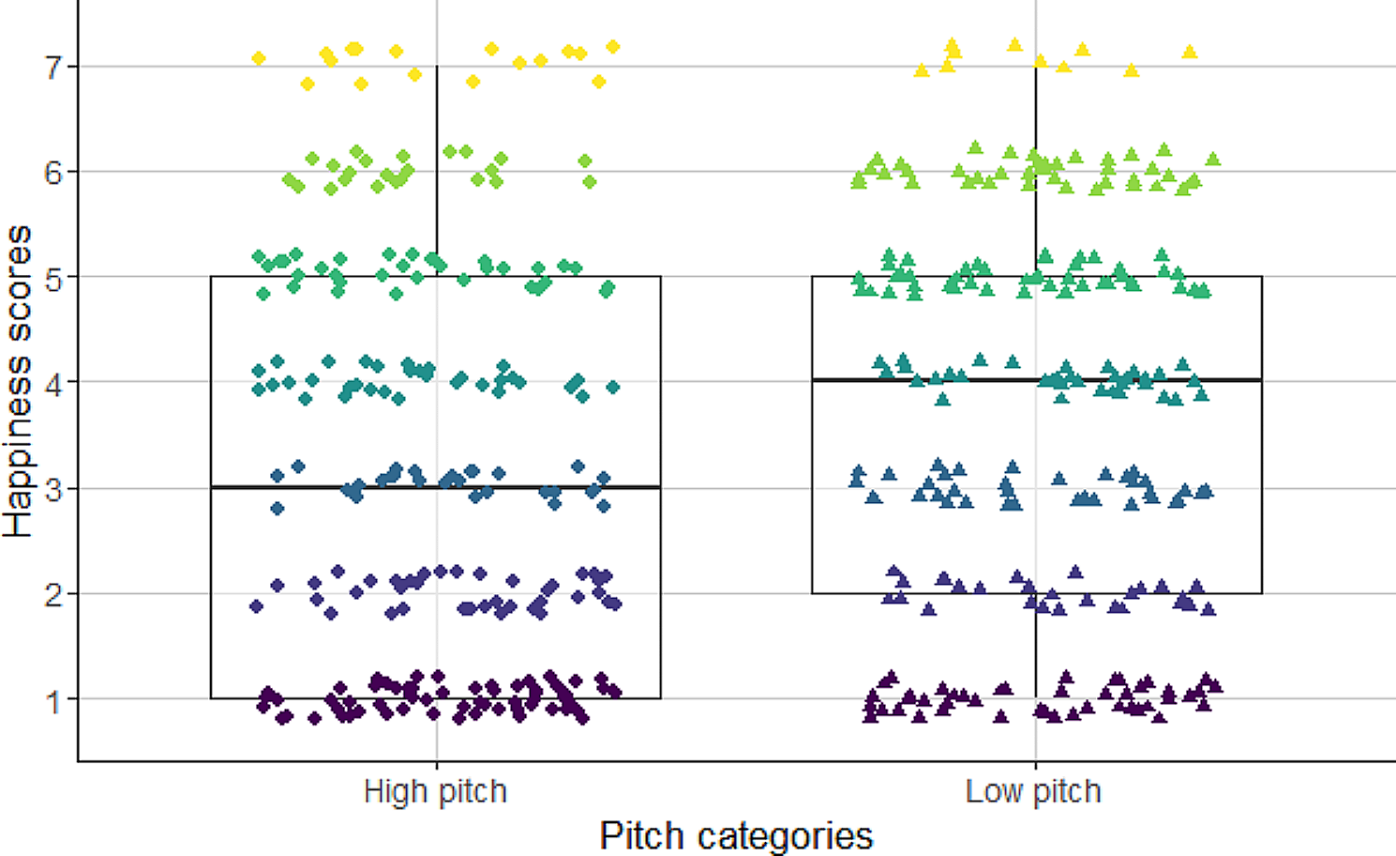
Association between the fundamental frequency of barks and the values given for “cheerful, playful” inner state scale. Barks with low fundamental frequencies seemed to be happier to the listeners

**Fig. 4:**
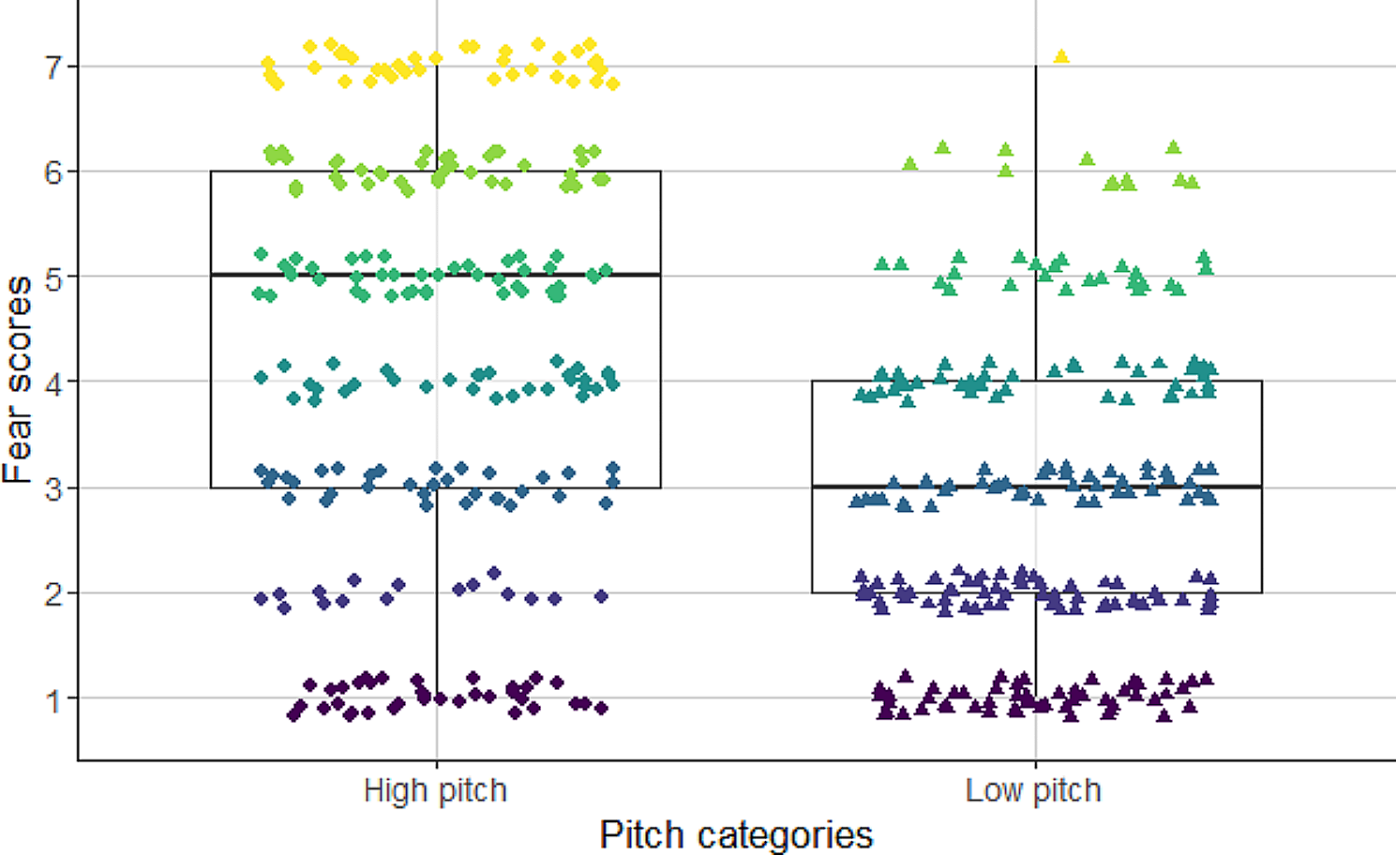
Association between fundamental frequency and fear as perceived inner state, evaluated by the participants. High fundamental frequency barks convey significantly more fear to the participants than low fundamental frequency barks

**Fig. 5:**
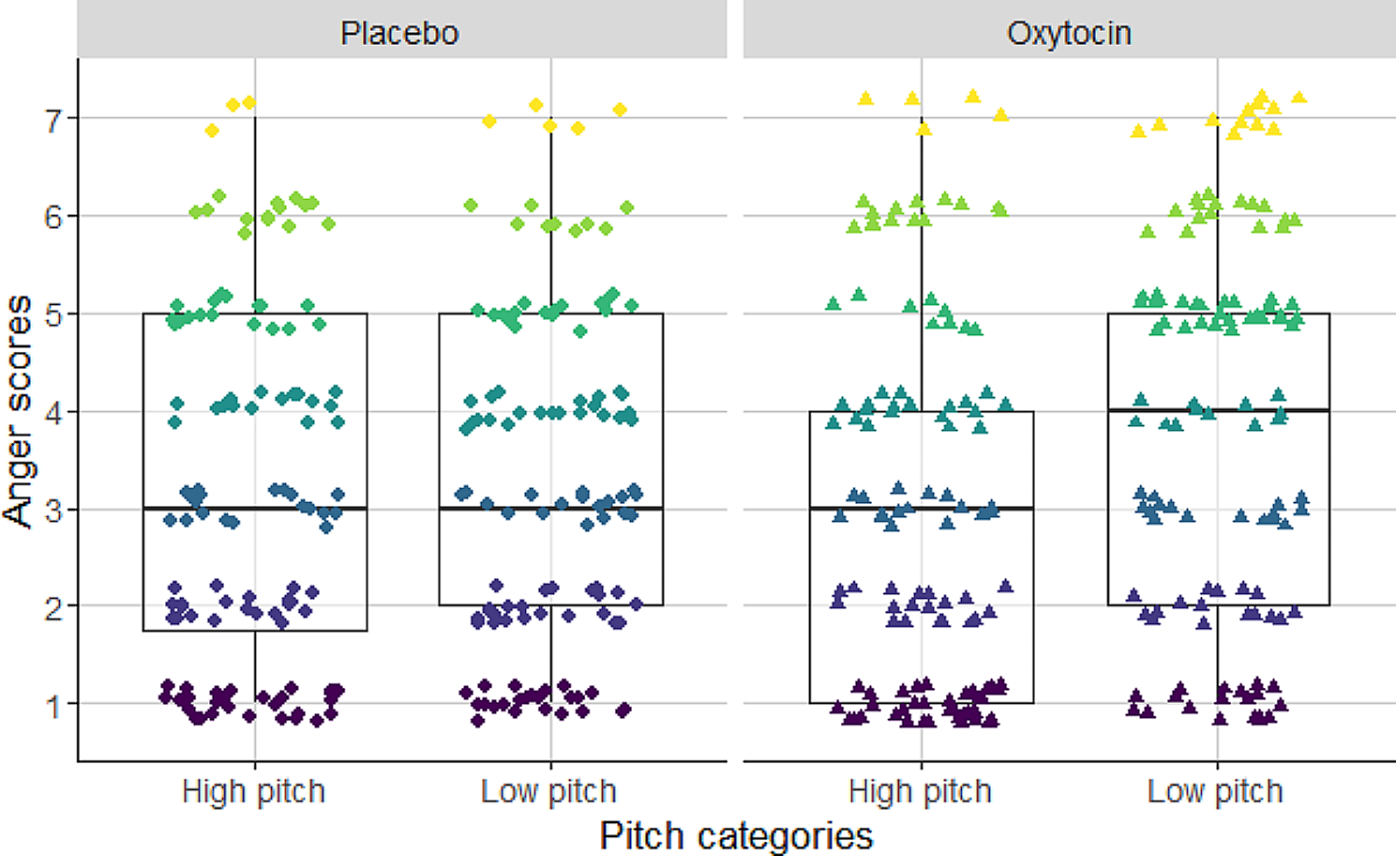
Two-way interaction between fundamental frequency and treatments. Low fundamental frequency barks were considered as being more aggressive than high fundamental frequency barks in the oxytocin-treated group. In contrast, there was no difference in the placebo-treated group between the fundamental frequency categories

We found a significant association between the fundamental frequency and the perceived emotional content of dog barks (see Figs. 3, 4 and 5; Table 3). Participants gave higher happiness scores to the low fundamental frequency barks than to high fundamental frequency barks (Fig. 3; Table 3). However, the intranasal oxytocin treatment had no significant effect on either the assessment of inner state ‘happy, playful’ or ‘desperate, fearful’.

**Table 3:** The main effect of fundamental frequency on ‘happiness’ rating

	Model 2: Positive emotion scores			
*Predictors*	*Odds Ratios*	*CI*	*Statistic*	*p*
pitch [Low]	1.487	1.078–2.051	2.418	**0.016**
**Random Effects**				
σ^2^	3.29			
τ_00 ID_	0.67			
ICC	0.17			
N _ID_	40			
Observations	480			
Marginal R^2^ / Conditional R^2^	0.010 / 0.177			

**Fig. 6:**
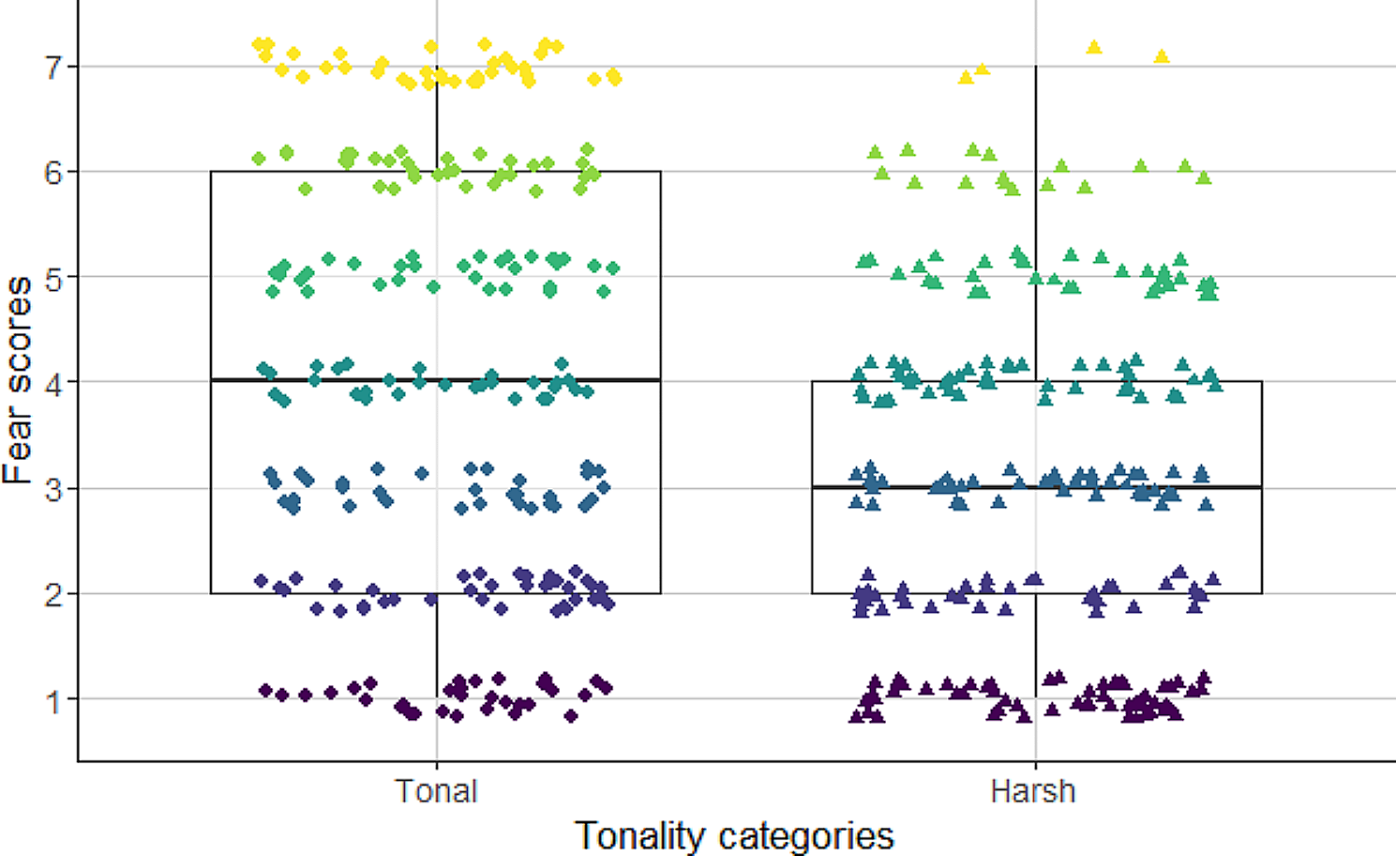
Association between tonality and fear, as the perceived inner state evaluated by the participants. Tonal barks convey significantly higher levels of fear to the participants, than atonal barks

Fear scores were also affected by the fundamental frequency and tonality (Table 4). Tonal barks with high fundamental frequency were considered as more fearful (Fig. 6) than low pitched and noisy, i.e., atonal barks (Fig. 4).

**Table 4:** The significant association between tonality and fundamental frequency with the assessment of fear

	Model 3: Fearful emotion scores			
*Predictors*	*Odds Ratios*	*CI*	*Statistic*	*p*
pitch [Low]	0.225	0.160–0.318	-8.487	**< 0.001**
tonality [Noisy]	0.392	0.283–0.544	-5.612	**< 0.001**
**Random Effects**				
σ^2^	3.29			
τ_00 ID_	0.26			
ICC	0.07			
N _ID_	40			
Observations	480			
Marginal R^2^ / Conditional R^2^	0.179 / 0.240			

**Fig. 7:**
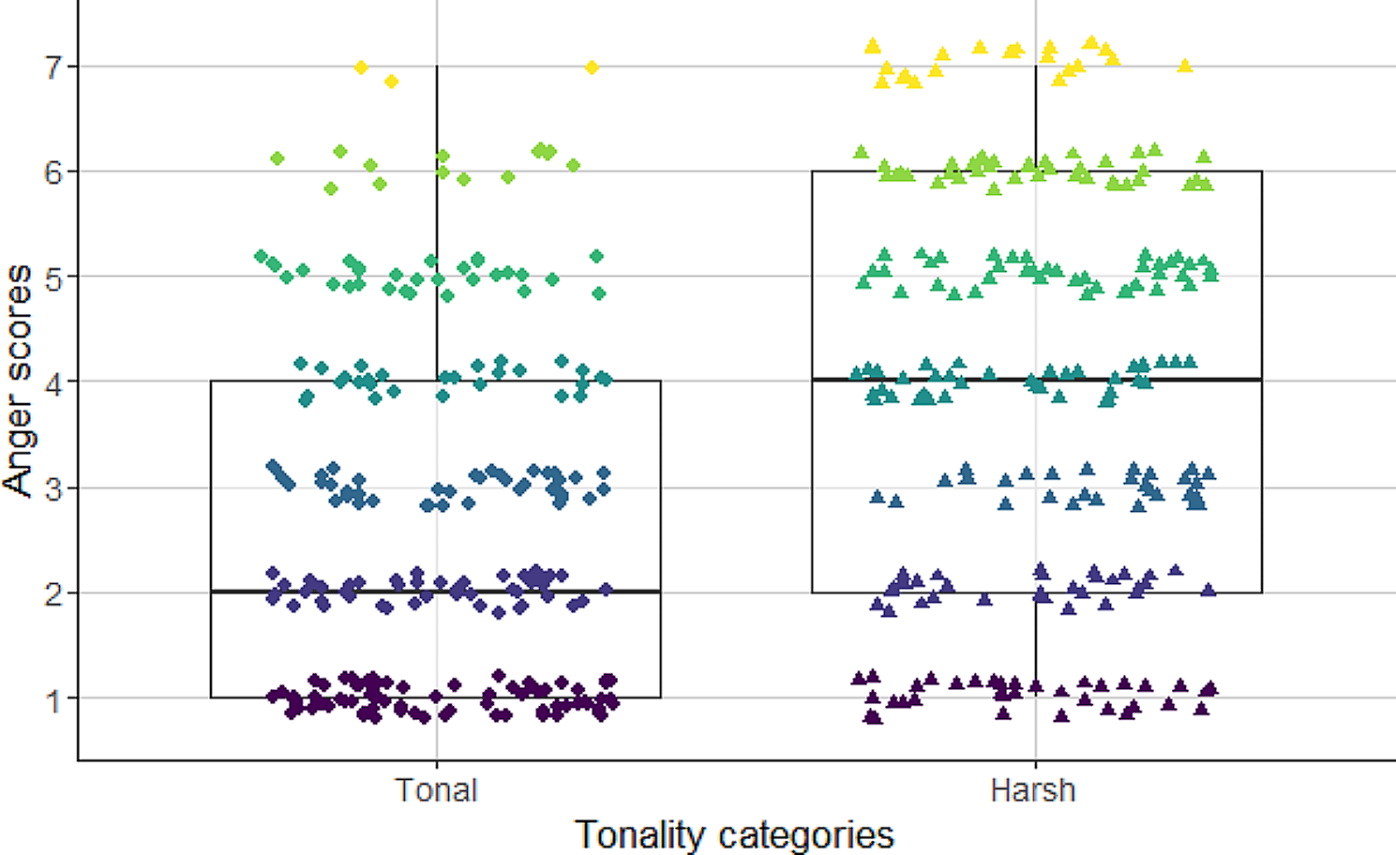
The association between scores of anger and the tonality of dog barks. While listening to the recordings, participants found atonal barks significantly angrier, than the tonal ones

Tonality had significant main effect on ‘angry, aggressive’ ratings (see Fig. 7; Table 5). Both treatment groups found noisy, atonal barks as being significantly angrier, than the high tonality ones. In addition, there was a two-way interaction between the treatments and the fundamental frequency (see Table 5). The Tukey’s Post-Hoc tests showed that participants treated with intranasal oxytocin perceived low-pitch barking as being more aggressive than the high-pitch barks (cum. prob ± SE = 0.152 ± 0.036; z = 4.172; *p* < 0.001; Fig. 5). In contrast, in the case of the participants who were treated with the placebo, we did not find significant association between the fundamental frequency of barks and the assessment of aggression (cum. prob ± SE = 0.016 ± 0.035; z = 0.464; *p* = 0.642).

**Table 5:** Significant effect of tonality and two-way interaction between treatment and fundamental frequency

	Model 4: Anger emotion scores			
*Predictors*	*Odds Ratios*	*CI*	*Statistic*	*p*
pitch [Low]	1.112	0.711–1.738	0.464	0.642
tonality [Noisy]	3.688	2.630–5.172	7.565	**< 0.001**
Treatment [Oxytocin]	0.832	0.413–1.678	-0.513	0.608
pitch [Low] × Treatment [Oxytocin]	2.399	1.254–4.587	2.645	**0.008**
**Random Effects**				
σ^2^	3.29			
τ_00 ID_	0.73			
ICC	0.18			
N _ID_	40			
Observations	480			
Marginal R^2^ / Conditional R^2^	0.123 / 0.283			

As a summary, it can be said that the intranasally administered oxytocin reduced the stress that the barks may have elicited in the participants. The subjects who received placebo treatment found the noisy barks to be more annoying, while in the case of the oxytocin-treated participants we found no difference in the annoyance scores based on the tonality of barks. However, oxytocin also had a sensitizing effect in the case of the perceived inner state of the dogs, connected to the association between the fundamental frequency of the barks and the aggression scores. The two treatment groups showed a notable difference in their assessment of dogs’ aggressiveness based on the fundamental frequency values of the barks.

## Discussion

In this study, we confirmed that particular acoustic features of dog barks may not only convey emotional information about the signaler [15], but they also affect the level of annoyance these barks elicit [30, 32]. Barks with high fundamental frequency values were given higher scores of fear, than low-pitch barks. Tonality also played an important role in the emotional evaluation of barks. Tonal (clear) barks were more likely to be found as being fearful, than atonal (noisy) ones. Thus for the listeners the emerging negative state conveyed by the barks was influenced by both the noisy and low fundamental frequency sounds. The scores of anger/aggression were negatively correlated with the fundamental frequency and the tonality of dog barks. The earlier found positive association between the high fundamental frequency and ‘happy, playful’ scores described in previous research [16, 32] was not confirmed by our current results. In contrast to our previous study, high-pitched barks were given very high ‘fear’ values, probably because when the participants had to evaluate the ‘happiness’, they were reluctant to provide high positive scores to the barks with high fundamental frequency values.

Fundamental frequency had a strong effect on the assessment of the elicited annoyance. Barks with high fundamental frequency were scored as being more annoying, than low fundamental frequency barks in both treatment groups. Similar to the findings of Pongrácz et al. [30], barks with mainly strong negative emotional state scores, received higher annoyance scores.

Based on a double-blind, placebo controlled intranasal oxytocin treatment for young adult male participants, we found support for both of our hypotheses. One of these hypotheses was that particular dog barks have a strong nuisance effect on human listeners through the (negative) emotions they convey. This hypothesis was confirmed with the result that those participants who received the oxytocin treatment, evaluated low fundamental frequency barks with higher aggression scores than the placebo-treated participants did. Thus, in the case of the connection between low-pitch barks, oxytocin had a sensitizing effect on the emotional understanding of the listeners. Based on our other hypothesis, we predicted that oxytocin will reduce the stress that may be caused by the alarming/attention calling function of particular barks. We confirmed this prediction with the results, which showed that participants who received placebo treatment found the atonal barks as being more annoying than the tonal barks (’tonality effect on annoyance’), while we did not find association between annoyance scores and the tonality in the case of participants who received intranasal oxytocin.

Previous research has shown that oxytocin increases the ability to recognize the inner state of another person. In the research of Guastella and colleagues [56], oxytocin treated participants remembered faces better that they had seen before, and they more easily recognized happy faces than angry or neutral ones. Evans and colleagues [57] detected that oxytocin reduced the aversion to anger seen on another person’s face. According to the results of the study by Domes et al. [39], intranasal oxytocin treatment made people more successful in recognizing another person’s mood based on a photo of the facial area around the eyes.

We provided the first indications of a dual effector system regarding the interspecific acoustic communication between dogs and humans. In our study it has been proven that oxytocin increased the ability of humans to assess the dog’s perceived inner state, especially emotions with negative valence, based on artificially assembled bark sequences. The ability of the listener to recognize and take into account the assumed emotional state based on dog barks plays a major role in the development of annoyance. However, at the same time, oxytocin attenuated the stress effect caused by (alarm) dog barking.

According to the two-way interaction between tonality and oxytocin treatment, it has been shown that placebo treated participants found noisy (atonal) barks more annoying, than tonal barks. However, in the case of the group treated with oxytocin, there was no such difference between tonal and atonal barks. It can be assumed that oxytocin-treatment detectably reduced the stress-enhancing and intervention-inducing effect [52–53] of nuisance barks [30, 51]. On the other hand, it is important to note that oxytocin only had an annoyance-reducing effect in the case of certain acoustic parameters. It modified the effect influenced by tonality, but it had no influence on the effect induced by fundamental frequency.

During the assessment of inner states, it has been found that as a function of fundamental frequency, participants who received oxytocin treatment gave higher anger scores to particular barks. As a result of oxytocin treatment, participants found low-pitched barks angrier than high frequency barks, therefore, anger is determined by tonality (noisier barks were found as being angrier) and frequency together. This result can be paralleled with the findings that in the case of vocal communication signals with an attention-eliciting function, e.g., baby cries [58], and low tonality (noisy) voices, are more likely to cause stress in people, than tonal (clear) voices.

Our current research confirmed that the most annoying dog barks also have a strong attention-grabbing role for humans. We can assume that these barks may signal such changes in the environment (i.e., a threat) that people would normally react to [16]. This could be one of the reasons why barks became the most variable and ubiquitous type of dog vocalizations during domestication [12], when intraspecific communication with humans was the new driving force behind the evolution of vocal signaling [12–14, 33].

There is acoustic similarity between a baby’s cry and dog barks [32, 59], and this is why these barks were coined as attention-grabbing barks. We conducted our research on that age group (potential parents) and sex (men) for whom barks with the specific attention-grabbing acoustic parameters were found to be the most annoying in earlier studies [30, 32]. Our research provides first-hand information, that in most cases, it is not the bark itself that bothers the listeners, but the dual components of stress and emotional reaction that particular barks may provoke.

Under the influence of such barks, listeners are urged to intervene and preferably change the situation that triggered the barking. A similar effect was described with baby cries [60]. If intervention is not possible (or unsuccessful), it induces frustration that causes stress and increases annoyance to the listener in the cases of baby cries [61] and dog barks (present study). The administration of intranasal oxytocin proved to be effective against developing higher levels of annoyance (i.e., frustration stress) in our participants; furthermore, it helped to perceive the (negative) emotional content of dog barks.

Our study has the limitation of using a male-only sample, which we opted for because earlier results showed a stronger nuisance effect of dog barks in young men than in other age cohorts and in women in general. Therefore, the results are not necessarily fully representative to other age classes and women. As dog owners are more likely female than male [62] and women show stronger emotional understanding towards animals as well [63], similar investigations would be worthy to be conducted on a more representative sample in the future.

## Conclusions

The coexistence of dogs and humans in crowded neighborhoods is often compromised by debates over nuisance barking. Our results emphasize that dogs can cause acoustic disturbance, not only because their barks are excessively abundant or loud, but also due to evolution causing dog barks to have specifically effective attention eliciting dual purpose attributes (informative-alarm and inner state-emotion), that causes humans to automatically want to investigate the reasons for the barking event. When there is a complaint issued about an excessively barking dog [51, 64], an ethological approach would be required to evaluate the situation, as well as registering the duration, volume and timing of the barking events. During the assessment, the situations when the dog most often barks (eliciting factors) should also be analyzed, as well as the acoustic parameters and unique characteristics of the barking event. In this way, humane corrective measures could be implemented to possibly avoid drastic solutions that involve negative consequences for the dog and its owner [65].

## Materials and methods

### Overall description and participants

Altogether 40 men, between 18 and 35 years of age, participated in our test. The control and the oxytocin-treated group included 20 male participants in each.

We made a playback test in which the participants were asked to listen to recordings of dog barks, which they had to assess one by one, with the help of scoring sheets. Before the test we included a two-phase pre-treatment. In the first phase, in a double blind procedure, we administered either oxytocin hormone or a placebo (NaCl solution) via intranasal spray. Following this treatment, we kept the participants isolated throughout the 40 min long incubation period (phase 2), to eliminate the chance that the results would be influenced by any external social effect. After the 40 min elapsed, they listened to the dog bark sequences.

**Fig. 8:**
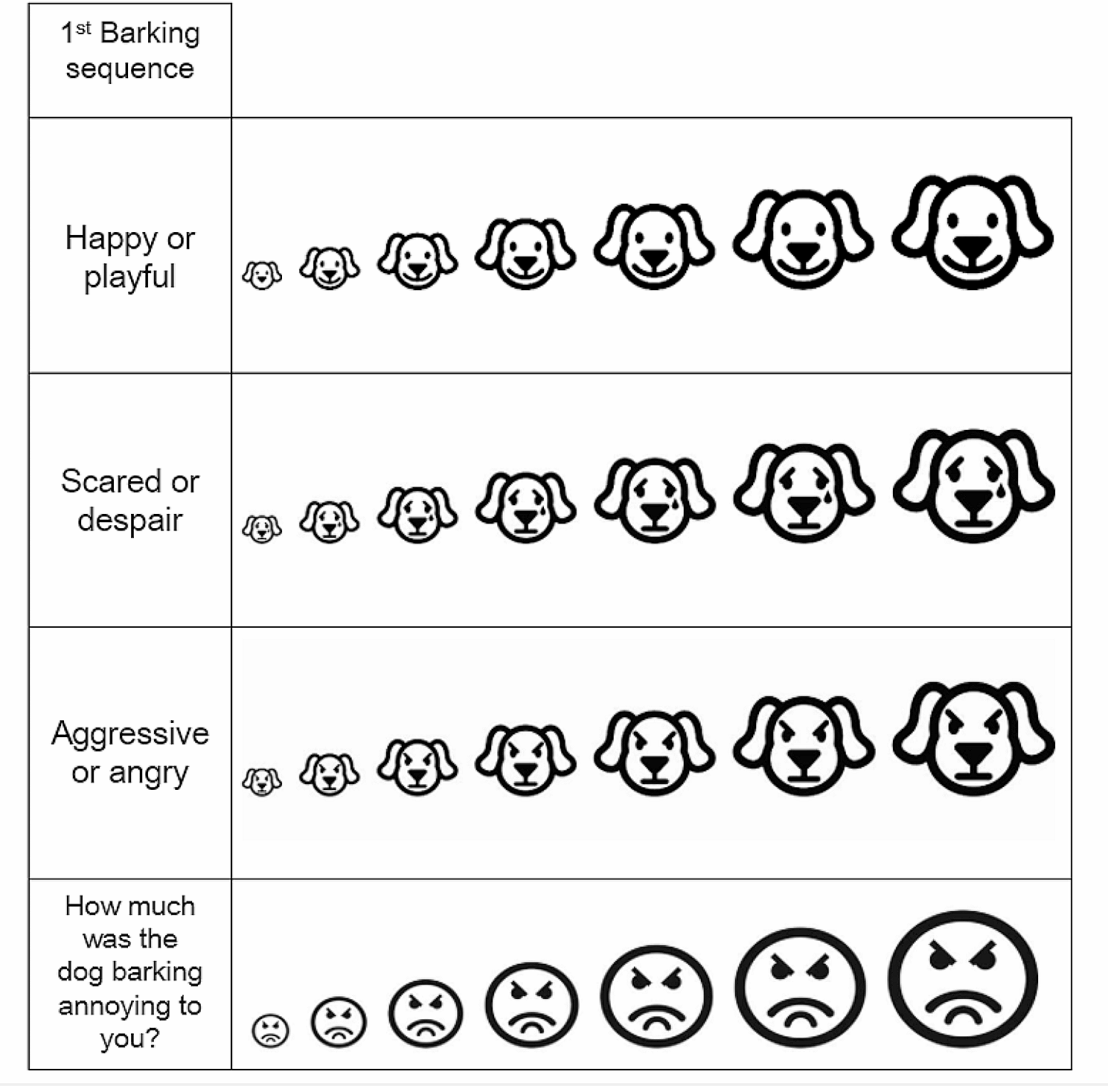
An excerpt of the scoring sheet that the participants used in the playback test. This is the sheet for the first recording in a playlist. The full set included one sheet for each of the 12 recordings. Based on [26]

Based on the bark samples, the participants had to evaluate the apparent inner states of the barking dogs and additionally, they had to rate each bark sequence according to the level of annoyance they triggered in the given participant. All ratings were done with the help of a Scoring Sheet (Fig. 8), on 7-grade Likert scales.

### Procedure

**Fig. 9:**
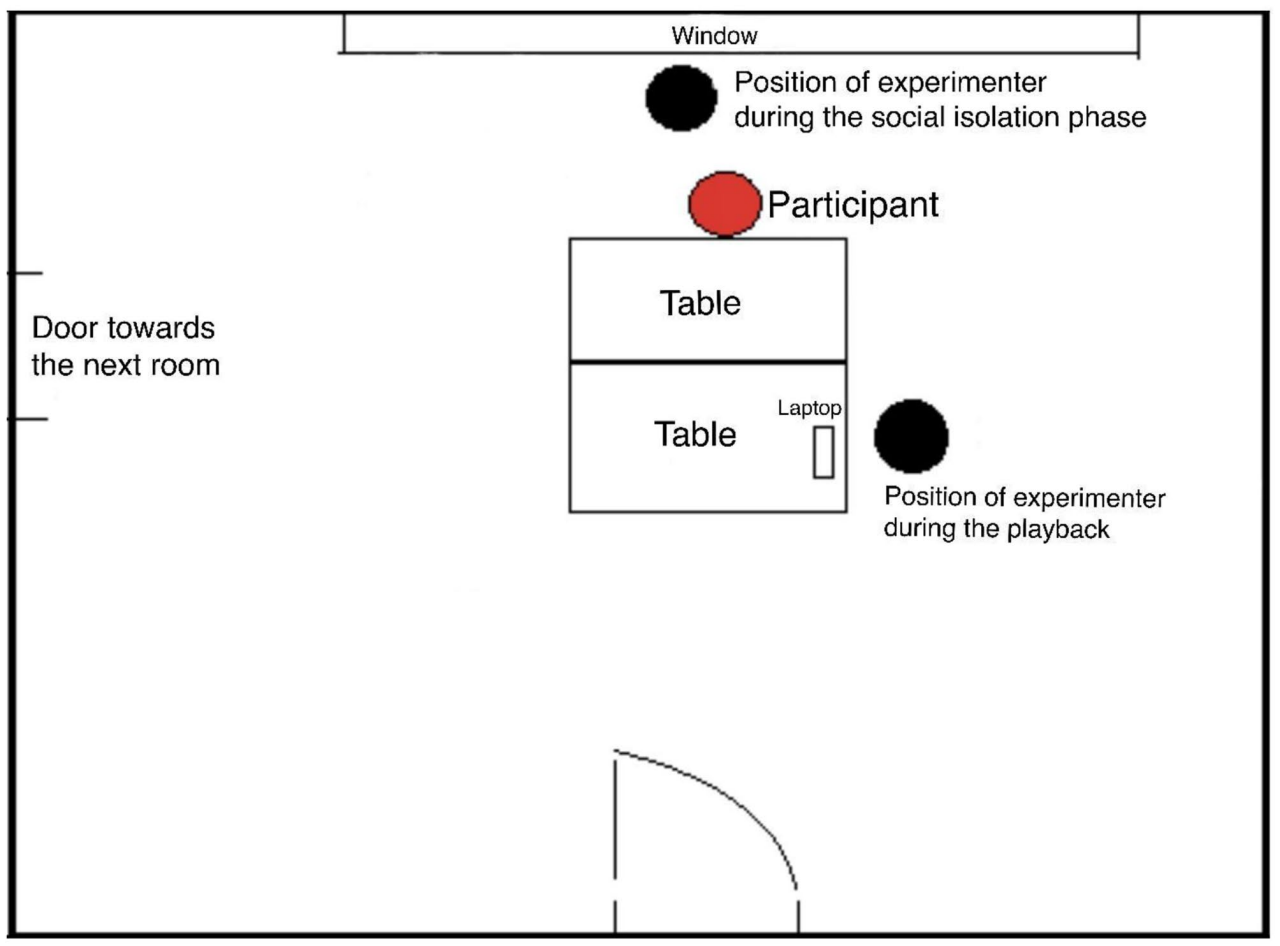
The schematic arrangement of the testing room

The experiment was conducted in the laboratory of the Department of Ethology (Fig. 9. shows the arrangement of the testing room). The whole test took 70 min, and each participant was tested only once. First, the participants received the informed consent form and the handout, which explained what the purpose of the study was and it also provided a short description of the experiment. After completing the consent form, the experimenter informed the participant about the procedure.

Next, we administered, via intranasal spray, the oxytocin hormone, or in the case of the control group, the placebo solution. Independently of their group-assignments, the participants were uniformly told that they were given oxytocin treatment. The nasal-spray bottles (10 ml) were identical in look between the oxytocin and control treatments, and could be identified only by their colored labels. Based on these labels, we created two groups, red and blue, that (unknown to the experimenter and participants) designated the oxytocin and placebo during the double blind procedure. The bottle with the red label contained oxytocin hormone, therefore the placebo was in the blue one. Filling of the bottles (with oxytocin (Syntocinon, Producer: Defiante Farmaceutica S.A., Germany) and with physiological NaCl solution used as placebo) was done by a third party, who did not participate in the study, so neither the experimenter, nor the participants, knew what the color codes meant. According to the storage instructions, the oxytocin (Syntocinon nasal-spray) should be kept cool (between + 2 and + 8 °C), so we kept both the control and oxytocin bottles refrigerated at the recommended temperature.

In all cases, participants were requested to perform the intranasal treatment (3–3 shots in both nostrils) for themselves. One spray shot contains approximately 4 IU (International Unit) oxytocin, therefore we used 24 IU/person. We chose this amount, as this is the most widely documented dosage in scientific literature [66–73]. The amount of oxytocin used in these studies is shown in Table 2.

Following the pre-treatment with the intranasal sprays, subjects had to wait 40 min in social isolation. Based on previous results this time frame of 40–45 min is necessary for the intranasal oxytocin treatment to reach effect [37, 40, 55, 66, 69, 71, 73, 74] with a maximum efficiency reached in 40 min [75]. During this time interval, the participants could not be affected by any external, social influences. Before the oxytocin pre-treatment, we asked them to turn off and put away every communication device, thus no social impact or interaction could happen on their end. During this isolation period they could not communicate with the experimenter either. Also, as demonstrated in Fig. 9, the experimenter was sitting behind the participant, therefore no social interaction could happen between them.

During the 40-minute isolation period the participants had to solve a 500-piece Ravensburger puzzle depicting a Mediterranean cityscape. There were no people depicted on the puzzle. The participants were previously told that they should solve this task at their own tempo. They were told that the puzzle was a part of the study, during which we do not evaluate the amount of solved puzzle pieces (thereby we were able to decrease the stress arising from the possibility of a competitive situation), only the strategy and colors they used were examined. We decided upon this task, because we wanted to occupy the participants with an action that had no social influence on them. After the 40-minute isolation we took a photo of the puzzle for documentation, imitating that it was a part of the study. Then the second main part began, which was the playback test.

### The playback test

During the playback test the participants listened to the bark recordings through noise-filtering Sennheiser headphones and a media player program (Winamp) in a closed, quiet room, where external noises did not disturb the test.

During the tests only the experimenter and the participant were present. All participants listened to a playlist including 12 bark sequences. The individual sequences were at least 3 and maximum 8 s long, depending on the interval between the bark units. The experimenter stopped the recording after each sequence, thus the participant could rate them one by one using the Likert scale (Fig. 8). The task was to evaluate the barks according to the assumed inner state of the dog (three separate scales for ‘happiness’, ‘fear’ and ‘anger’) and the annoyance level the barking triggered in the participant. Every sequence was typically played only once, but upon request by the participant, the experimenter could play it one more time.

### The scoring sheet

We asked the participants to rate each bark sequence individually. Besides the annoyance ratings, they also had to assess the inner state of the dog.

As these recordings were created artificially, in reality, the participants did not evaluate the inner state of a particular dog/sequence. Instead, they rated the perceived emotional state, based on the acoustic parameters of the assembled bark sequences. We provided the participants with individual scoring sheets for each bark sequence (Fig. 8) with three questions regarding the dog’s inner state (‘Happy, playful’, ‘Scared, desperate’, ‘Aggressive, angry’) and one question regarding the degree of annoyance triggered by the barking (”How annoying was this dog barking to you?”). Next to each of the questions they could find a scoring scale, which was a modified Likert scale with stylized dog and human faces, which in turn represented the values of the scale. Linearly increasing, the smallest picture represented the ‘weakest’ and the largest indicated the ‘strongest’ value on the given scale. The scoring scales about the dog’s emotional state were illustrated by stylized dog faces showing different emotions, while the scale indicating the participant’s annoyance elicited by the given bark sequence was pictured with annoyed human faces. This scoring sheet was based on a scoring system created for children in previous research [18]. In this study we used the same sheet as in our previous study [32], because we wanted to use a standardized method for our new experiment, which was based on our previous findings. From the scoring sheets the participants’ responses were entered to a database in a digitalized format for further analysis.

### The sound samples

We used artificially assembled sequences of dog barking events, which were created from original recordings taken during field work [16] in different contexts. The bark recordings were at first segmented to individual bark units, then with a computer-based algorithm the new artificial sequences [30] were created. We used artificially assembled sequences, because this way we could control those acoustic characteristics that would impact what we intended to investigate. Also, we were able to exclude the individual characteristics of the barking dogs and the well-recognizable acoustic characteristics of context-specific barks [15, 30]. In the pool of bark units, we had recordings from 26 different dogs, all of them were from the Mudi breed. This herding breed is strongly vocal as a result of their original function. The original bark sequences were recorded in six different social contexts during a previous study (the methodological description about the process of the recording is accessible: [16]. The situations in which the recordings were taken are: “Stranger at the fence”, “Schutzhund/Fight training”, “(left) Alone”, “Before walk”, “(asking for) Ball”, “Play with owner” (for detailed description of the situations see: [16]). For selecting the bark units of the artificially created bark sequences, we based our choice on two categories (low and high) of tonality and fundamental frequency in the case of each unit. From the original recordings 1452 bark units were selected, based on their tonality (Harmonic to Noise Ratio (HNR): low: -2.1–4.6, high: 11.6–35.4) and pitch (fundamental frequency: low: 401–531 Hz, high: 732–1883 Hz).

Based on the previous research results [30], we excluded the ’medium’ values from the tonality and pitch samples, because in the original study these did not have a significant effect on annoyance ratings. This allowed retention of the most and the least annoying bark sequences based on the study of Jégh-Czinege et al. [32]. From the selected bark units we created artificial bark sequences (with 10 individual bark units in each). For the assembly of the bark sequences we used three categories of between-bark time intervals (short: 0.1 s, medium: 0.3 s, long: 0.5 s long break).

As a result, we ended up with 12 types (2 × 2 × 3) of bark sequences (Table 6). We created playlists from these, each containing only one from each of the 12 sequence types in random order. We only used each playlist once, so every participant listened to a different playlist, this way we could avoid the effect of pseudoreplication in our study.

**Table 6:** The 12 artificially assembled bark sequences. Abbreviations: p = pitch (F0), h = HNR (tonality), i = interval (time length between each bark unit). Hi = high, med = medium, low = low

F0 (pitch)	tonality	Interbark interval
hip	hih	hii
hip	hih	lowi
hip	hih	medi
hip	lowh	hii
hip	lowh	lowi
hip	lowh	medi
lowp	hih	hii
lowp	hih	lowi
lowp	hih	medi
lowp	lowh	hii
lowp	lowh	lowi
lowp	lowh	medi

### Statistical analysis

All statistical analyses were performed with R Studio (RCoreTeam, 2017). We used Cumulative Link Mixed Models fitted with the Laplace approximation (ordinal package, clmm function) to investigate which factors influenced the scoring of the dogs’ inner states and annoyance scores that were elicited by the dog barks in the participants. Oxytocin or placebo treatments, and acoustic parameters (the high and low levels of fundamental frequency and tonality, besides three levels of inter-bark intervals: short, medium, long) were used as independent factors. Two way interactions of Treatment and acoustic categories were also included in the initial models. ID of the participants was used as random factor. On the initial models we ran AIC-based backwards model selection (drop1 function). During this, the effects contributing least to the model fit were eliminated one by one from the model until the simplest, yet best-fitting model was obtained We run Tukey’s Post-Hoc test for pairwise comparisons (emmeans package, emmeans function).

### Electronic supplementary material

Below is the link to the electronic supplementary material.

**Supplementary Material 1:** Sound sample of a bark sequence, containing individual barks of low pitch, low tonality, assembled with short interbark intervals in between

**Supplementary Material 2:** Sound sample of a bark sequence, containing individual barks of high pitch, high tonality, assembled with long interbark intervals in between

**Supplementary Material 3: Table S1.** Raw data used for statistical analysis

## Data Availability

All data generated or analyzed during this study are included in this published article [and its supplementary information files]. Table S1 shows the raw data used for the analyses.
